# Cavitary Pulmonary Blastomycosis and an Incidental Retroperitoneal Ganglioneuroma Mimicking Metastatic Disease

**DOI:** 10.7759/cureus.103664

**Published:** 2026-02-15

**Authors:** Matthew T Foley, Kaylin M Burton, Alec McKheen, Matthew Karulf

**Affiliations:** 1 Internal Medicine, Michigan State University College of Human Medicine, East Lansing, USA; 2 Anesthesiology, Michigan State University College of Human Medicine, East Lansing, USA; 3 Pulmonology, Upper Peninsula Health System-Marquette, Marquette, USA

**Keywords:** blastomycosis, cavitary lung disease, fungal infection, ganglioneuroma, pulmonary blastomycosis, retroperitoneal mass

## Abstract

Pulmonary blastomycosis is a fungal infection caused by *Blastomyces dermatitidis* that can present with a wide range of nonspecific pulmonary findings and, in rare cases, mimic metastatic malignancy. This case report describes a 26-year-old immunocompetent male who presented with subacute respiratory symptoms and was found to have numerous cavitary pulmonary lesions and a large infiltrative retroperitoneal mass, raising concern for disseminated malignancy, with subsequent evaluation revealing pulmonary blastomycosis and an incidental benign retroperitoneal ganglioneuroma.

## Introduction

Blastomycosis is a fungal infection caused by inhalation of Blastomyces dermatitidis, an organism endemic to regions including the Ohio and Mississippi River Valleys, the Great Lakes region, and parts of the southeastern United States [1]. Environmental exposure to disturbed soil has been strongly associated with acquisition of blastomycosis, particularly in endemic areas [2]. Pulmonary involvement is the most common manifestation and often presents with nonspecific symptoms such as cough, fever, chills, dyspnea, and chest pain, frequently leading to delayed diagnosis or misdiagnosis as bacterial pneumonia or malignancy [3].

Radiographic findings in pulmonary blastomycosis are variable and may include consolidations, nodules, masses, or cavitary lesions, which can closely resemble metastatic disease or septic emboli [4]. Ganglioneuroma is a rare, benign neural crest-derived tumor most commonly located in the posterior mediastinum or retroperitoneum. Although benign, its imaging appearance may mimic malignant retroperitoneal neoplasms, particularly when large or encasing adjacent vascular structures [5].

We describe a young immunocompetent male who presented with cavitary pulmonary lesions and a large retroperitoneal mass initially concerning for disseminated malignancy, ultimately diagnosed with pulmonary blastomycosis and an incidental benign neural tumor. This case highlights a potential diagnostic pitfall in which concurrent infectious and incidental neoplastic processes create imaging findings highly suspicious for metastatic disease, underscoring the importance of tissue confirmation prior to definitive management decisions.

## Case presentation

A 26-year-old male with no significant past medical history presented with a three-week history of fevers, chills, malaise, pleuritic chest discomfort, and a productive cough with thick yellow and green sputum. Several family members and coworkers experienced similar respiratory symptoms that resolved spontaneously while his symptoms persisted.

He was referred for hospital admission following abnormal outpatient computed tomography (CT) of the chest revealing diffuse cavitary pulmonary nodules with a broad differential diagnosis. 

Prior to symptom onset, the patient reported performing construction and excavation work during which time he worked continuously in a dense cloud of soil and dust without any mask or personal protective equipment, which may have been a nidus for infection. He also reported frequent vaping of high-concentration nicotine products. He denied weight loss, recent travel, intravenous drug use, sick contacts, or known tuberculosis exposure. Initial laboratory results upon admission are shown in Table 1. 

**Table 1:** Initial laboratory results upon admission from outside hospital. Values: "H" = elevated value, "L" = low value, "–" = within reference range. MRSA = methicillin-resistant Staphylococcus aureus

Component	Value	Reference Range	Flag
White Blood Cell Count (WBC)	18.7 K/µL	4.0 – 11.0 K/µL	H
Hemoglobin (Hgb)	11.8 g/dL	13.5 – 17.5 g/dL	L
Platelet Count (Plt)	799 K/µL	150 – 450 K/µL	H
Erythrocyte Sedimentation Rate (ESR)	81 mm/hr	0 – 15 mm/hr	H
C-Reactive Protein (CRP)	6.4 mg/dL	< 0.5 mg/dL	H
D-dimer	2.68 mg/L	< 0.50 mg/L	H
Procalcitonin	0.181 ng/mL	< 0.10 ng/mL	H
SARS-CoV-2 PCR	Negative	Negative	–
Influenza A/B	Negative	Negative	–
Respiratory Syncytial Virus (RSV)	Negative	Negative	–
Respiratory Viral Panel	Negative	Negative	–
HIV-1 RNA	Not Detected	Not Detected	–
MRSA Nares Screen	Negative	Negative	–
Sputum Culture	Haemophilus parainfluenzae	Normal flora	Abnormal

**Figure 1:**
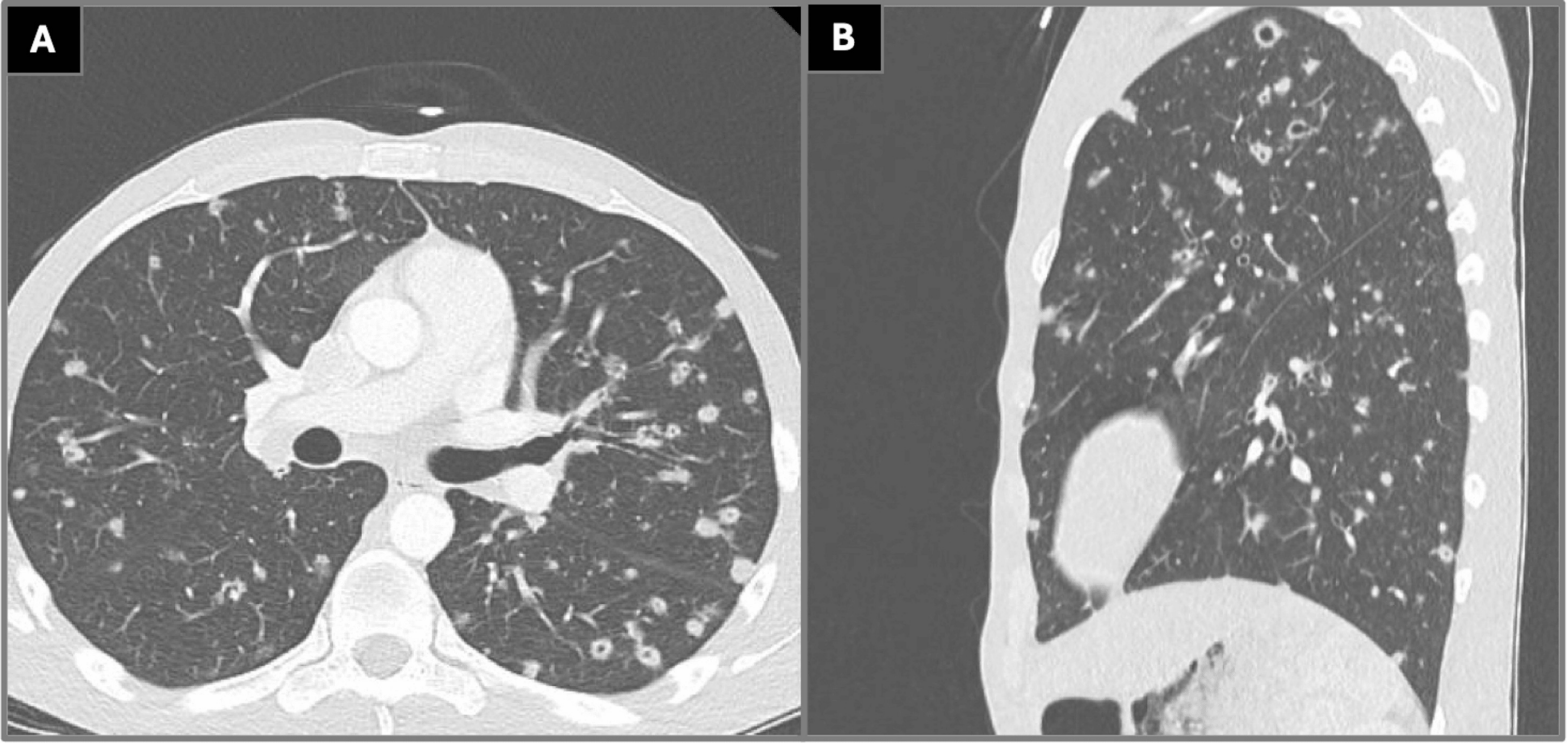
Computed tomography of the chest demonstrating numerous cavitary pulmonary nodules involving multiple lobes. (A) Axial lung window showing multiple irregular cavitary lesions bilaterally. (B) Sagittal reconstruction illustrating the diffuse distribution of cavitary nodules throughout the lung parenchyma, findings consistent with pulmonary blastomycosis.

**Figure 2:**
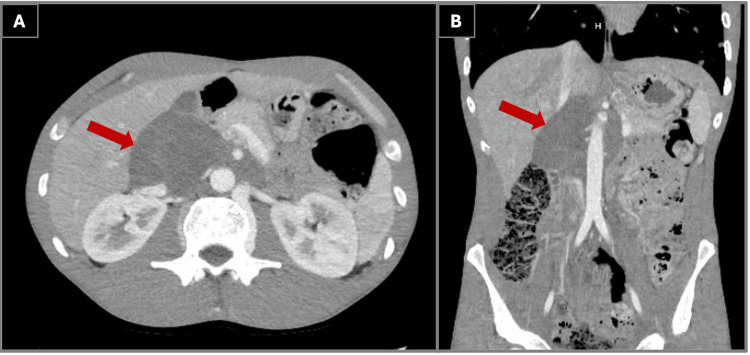
Contrast-enhanced computed tomography of the abdomen demonstrating a large infiltrative retroperitoneal mass. (A) Axial view revealing a hypodense retroperitoneal soft-tissue mass encasing the abdominal aorta and inferior vena cava. (B) Coronal reconstruction illustrating the craniocaudal extent of the mass with encasement of major mesenteric vessels, imaging features initially concerning for malignancy and subsequently confirmed as a benign ganglioneuroma on histopathology.

CT imaging of the chest demonstrated numerous cavitary pulmonary nodules involving all lobes, with the largest lesion measuring approximately 3.6 cm, as shown in Figure 1. A partially visualized upper abdominal mass was incidentally noted on this study, prompting dedicated contrast-enhanced CT imaging of the abdomen and pelvis. Follow-up imaging of this mass revealed a large infiltrative retroperitoneal mass measuring approximately 5.9 × 9.1 × 11.9 cm, hypodense in attenuation, encasing the inferior vena cava and aorta with involvement of the superior mesenteric artery and right renal artery and partial involvement of the celiac trunk and left renal artery, as shown in Figure 2. These findings were highly concerning for metastatic malignancy or possibly septic emboli.

Given the suspicion for infective endocarditis, transthoracic and transesophageal echocardiography were obtained which did not reveal any evidence of endocarditis. Given concern for possible lymphoma, a testicular ultrasound was obtained and did not reveal any evidence of a testicular mass. Tumor markers including alpha-fetoprotein and lactate dehydrogenase were unremarkable.

**Figure 3:**
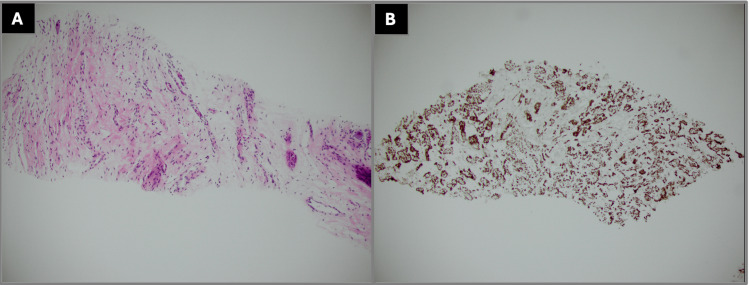
Histological characterization of retroperitoneal mass biopsy. (A) Histology of retroperitoneal mass biopsy revealing spindle cells with elongated, wavy nuclei in a myxocollagenous background with scattered ganglion cells. (B) Immunohistochemical stain showing positive S100 expression of spindle cells

Interventional radiology was consulted and the patient underwent CT-guided core needle biopsy of the retroperitoneal mass, with histological evaluation revealing a lesion composed of spindle cells with elongated, wavy nuclei in a myxocollagenous background with scattered ganglion cells. Immunohistochemical staining demonstrated S100 positivity, as shown in Figure 3. Staining for smooth muscle actin and desmin was negative. Histology did not reveal any cytologic atypia, mitotic activity, or necrosis. Overall histology was consistent with a ganglioneuroma.

**Figure 4:**
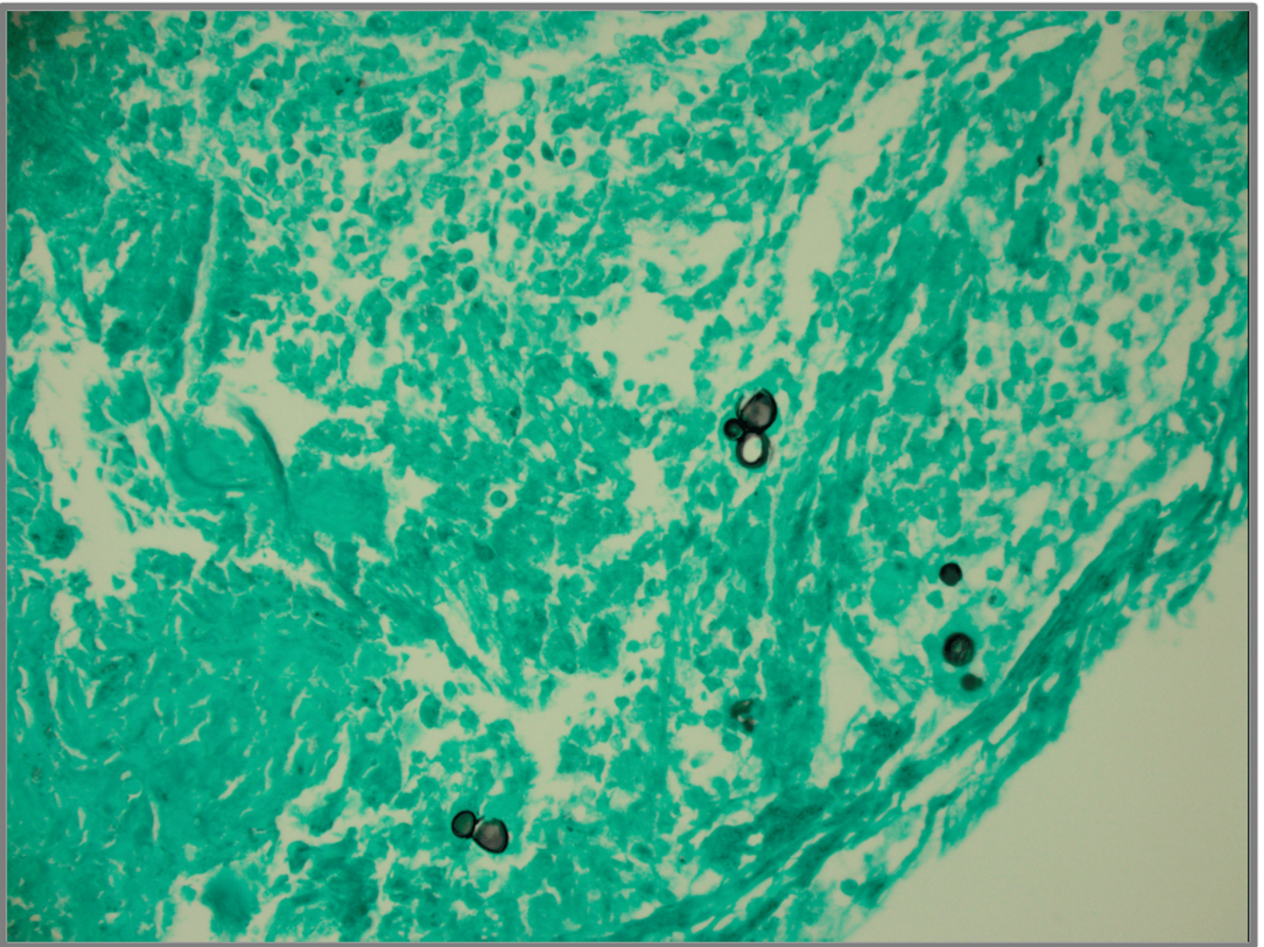
Histology of right upper lobe lung biopsy with Grocott’s Methenamine Silver stain revealing yeast forms with broad based budding, compatible with Blastomycoses.

Pulmonology was consulted, and diagnostic bronchoscopy with bronchoalveolar lavage, transbronchial biopsy, and endobronchial ultrasound-guided transbronchial needle aspiration was performed. Histopathologic examination of right upper lobe lung biopsy specimens demonstrated mixed inflammation including histiocytes, multinucleated giant cells, and acute exudate with fungal organisms identified, consistent with pulmonary blastomycosis as shown in Figure 4. 

The patient was treated initially with two days of intravenous (IV) cefepime 2 grams every 24 hours and later was de-escalated to IV ceftriaxone 2 grams every 24 hours for seven days during the hospital for Haemophilus parainfluenzae pneumonia. The patient was also treated with itraconazole 100mg twice daily for 30 days for pulmonary blastomycosis with refills available upon follow-up with primary care physician or infectious disease. Given the absence of respiratory failure, central nervous system involvement, or immunocompromising conditions, amphotericin B therapy was not indicated in accordance with current guideline-based management. He demonstrated rapid clinical improvement, remained afebrile, and maintained adequate oxygenation on room air prior to discharge.

The patient was initially seen by infectious disease via telehealth shortly after discharge and continued oral itraconazole therapy. However, he was subsequently lost to follow-up, and no repeat imaging or long-term clinical outcome data are available within our system.

## Discussion

Pulmonary blastomycosis is an endemic fungal infection with a broad spectrum of clinical and radiographic presentations, often mimicking bacterial pneumonia, tuberculosis, or malignancy [3]. Cavitary pulmonary lesions, while less common, may represent more severe pulmonary involvement and contribute to significant diagnostic uncertainty, particularly when extrapulmonary findings are present [6].

In this case, the presence of numerous cavitary pulmonary nodules in conjunction with a large infiltrative retroperitoneal mass prompted extensive evaluation for disseminated malignancy. Retroperitoneal involvement in blastomycosis is uncommon, and imaging findings alone are insufficient to reliably distinguish infectious from neoplastic processes [7]. 

Ganglioneuromas are rare benign tumors arising from neural crest cells and are most commonly located in the posterior mediastinum and retroperitoneum [8]. These tumors are often asymptomatic and discovered incidentally during imaging for unrelated conditions. Although benign, their size and infiltrative appearance may closely resemble malignant processes on cross-sectional imaging [9].

The coexistence of pulmonary blastomycosis and an incidental retroperitoneal ganglioneuroma in this patient underscores the potential for coincidental pathology to complicate diagnostic evaluation. Histopathologic confirmation was essential in distinguishing these two processes and guiding appropriate management. This case highlights the importance of histologic evaluation in patients with complex radiographic findings, particularly when imaging suggests disseminated malignancy in the setting of concurrent but unrelated pathology. Premature diagnostic anchoring in such scenarios may lead to unnecessary invasive procedures or inappropriate oncologic treatment.

## Conclusions

This case illustrates the diagnostic challenges posed by pulmonary blastomycosis presenting with cavitary lung disease, particularly when imaging findings raise concern for disseminated malignancy. Incidental radiological findings such as ganglioneuroma may further confound clinical assessment. Definitive diagnosis via biopsy and histologic evaluation remains critical in complex cases to avoid misdiagnosis and ensure appropriate treatment. Early recognition of blastomycosis and prompt initiation of antifungal therapy are critical in preventing progression of disease and avoiding misdiagnosis as malignancy. This case underscores the diagnostic complexity that may arise when infectious and incidental benign processes coexist and reinforces the necessity of tissue confirmation prior to definitive management decisions.
